# Susceptibility to Fear of Heights in Bilateral Vestibulopathy and Other Disorders of Vertigo and Balance

**DOI:** 10.3389/fneur.2018.00406

**Published:** 2018-06-06

**Authors:** Thomas Brandt, Eva Grill, Michael Strupp, Doreen Huppert

**Affiliations:** ^1^German Center for Vertigo and Balance Disorders, Ludwig-Maximilians-University, University Hospital, Munich, Germany; ^2^Institute for Clinical Neurosciences, Ludwig-Maximilians-University, University Hospital, Munich, Germany; ^3^Institute for Medical Information Processing, Biometrics and Epidemiology, Ludwig-Maximilians-University, Munich, Germany; ^4^Department of Neurology, Ludwig-Maximilians-University, University Hospital, Munich, Germany

**Keywords:** bilateral vestibulopathy, vestibular migraine, Menière's disease, benign paroxysmal positional vertigo, phobic postural vertigo, visual height intolerance

## Abstract

**Aims:** To determine the susceptibility to visual height intolerance (vHI) in patients with acquired bilateral vestibulopathy (BVP). The question was whether postural instability in BVP, which is partially compensated for by visual substitution of the impaired vestibular control of balance, leads to an increased susceptibility. This is of particular importance since fear of heights is dependent on body posture, and visual control of balance at heights can no longer substitute vestibular input. For comparison susceptibility to vHI was determined in patients with other vestibular or functional disorders.

**Methods:** A total of 150 patients aged 18 or above who had been referred to the German Center for Vertigo and Balance Disorders and diagnosed to have BVP were surveyed with a standardized questionnaire by specifically trained neurological professionals. Further, 481 patients with other vestibular or functional disorders were included.

**Results:** Susceptibility to vHI was reported by 29% (32 % in females, 25% in males) of the patients with BVP. Patients with vHI were slightly younger (67 vs. 71 years). Seventy percent of those with vHI reported avoidance of climbing, hiking, stairs, darkness, cycling or swimming (84% of those without vHI). Mean age for onset of vHI was 40 years. Susceptibility to vHI was higher in patients with other vertigo disorders than in those with BVP: 64% in those with phobic postural vertigo, 61% in vestibular migraine, 56% in vestibular paroxysmia, 54% in benign paroxysmal positional vertigo, 49% in unilateral vestibulopathy and 48% in Menière's disease.

**Conclusions:** The susceptibility to vHI in BVP was not higher than that of the general population (28%).This allows two explanations that need not be alternatives but contribute to each other: (1) Patients with a bilateral peripheral vestibular deficit largely avoid exposure to heights because of their postural instability. (2) The irrational anxiety to fall from heights triggers increased susceptibility to vHI, not the objective postural instability. However, patients with BVP do not exhibit increased comorbid anxiety disorders. This view is supported by the significantly increased susceptibility to vHI in other vestibular syndromes, which are characterized by an increased comorbidity of anxiety disorders.

## Introduction

### Historical view on mechanisms of fear of heights associated with postural imbalance

A short historical excursus may be permitted here as to the dependence of fear of heights on posture, locomotion, and balance control, all of which are affected by bilateral vestibulopathy. There is phylogenetic evidence of an inborn behavioral pattern allowing us to perceive and avoid heights, the so-called “visual cliff” phenomenon. This broadly gene-linked avoidance of depths in order to prevent falls off cliffs was comprehensively studied by Walk et al. (1), Walk and Gibson (2). The innate ability of pre-walking and walking infants to visually avoid a brink (3, 4) was further supported by animal experiments in a number of other species including chicks, rats, kittens, and goats. There are two visual signs for perceiving height as a special case of distance: the drop-off increases in texture density beyond the edge, and the absolute and relative motion parallax cues differ during active locomotion and head movements. Both texture/density preferences (5, 6) and motion parallax cues are utilized and interact with each other.

Therefore, it is not surprising that our ancestors were well aware of visual height intolerance. There are numerous descriptions of provoking situations and typical symptoms in the Greek, Roman, and Chinese classics which suggest visual height intolerance and fear of heights (7). One example is found in Titus Livius's history on the second Punic war, where he describes how soldiers on high ladders plunged to the ground when attacking Carthago Nova because the heights “had veiled their eyes with dizziness.” Another example is Hannibal's crossing of the Alps in the third century BC. Silius Italicus recounted this feat in *Punica*, describing how “the gaze became dizzy on the high rocks” (8). In the Chinese classic *Huangdi Neijing*, the Yellow Thearch is said to suffer from a confusion and to feel dizzy when he climbed up on to an observation platform (9). It is interesting that several historical sources report stance and gait being disturbed. Demokles, for example, experienced a “slackening of the muscles of the entire body” (Greek *Corpus Hippocraticum* fifth century BC) when walking along the edge of a precipice or over a bridge; however, he would dare to walk in the ditch itself. Similarly Phaeton turned pale while driving the sun chariot (Ovid's *Metamorphoses*, about 0–8 AD) and suddenly felt his knees trembling from fear (8). Later the main ouvre of Erasmus Darwin, *Zoonomia, or The Laws of Organic Life* in 1794 (10), also provided a detailed early concept of disturbed sensorimotor control when exposed to heights. The Yellow Thearch in ancient China intuitively found a treatment, namely kneeling on the ground: it alleviated the symptoms that had occurred at heights while he stood on an observation platform. This historical “antidote” corresponds to contemporary psychophysical experiments (11, 12).

**Figure 1:**
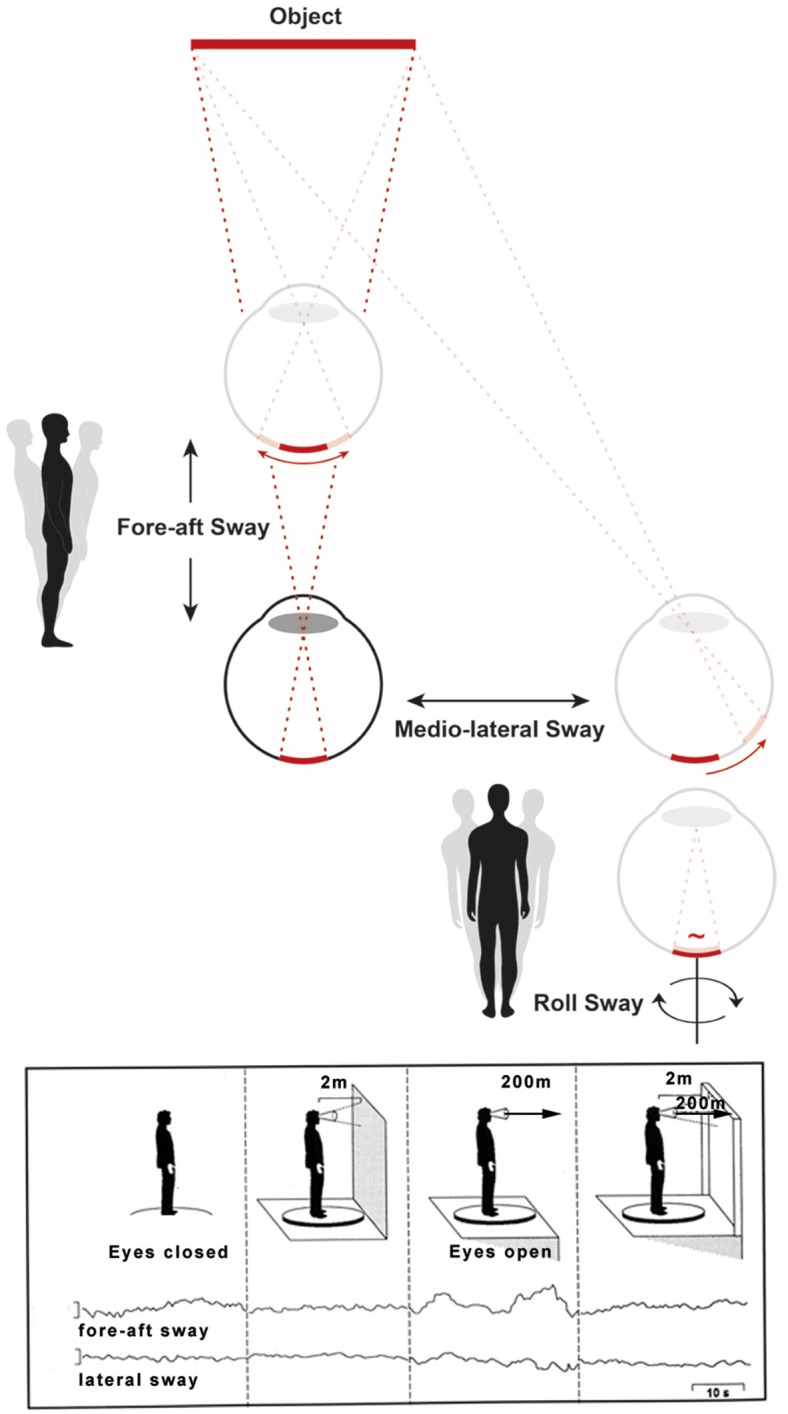
The mechanism of a physiological postural imbalance at visual heights can be explained by the dependence of the retinal slip of viewed objects on their distance. This is depicted schematically **(Top)**. Visual control of body sway in fore-aft, lateral, and roll planes shows that angular displacement on the retina caused by fore-aft and lateral head displacements are smaller, the greater the distance is to the object. Therefore, when exposed to heights, the head sway goes visually undetected (the retinal slip is below the threshold value for detecting motion) and thus impairs visual stabilization of posture. This is different for head and body sway in the roll plane **(Bottom** right of the **Top** figure). In this plane, the distance between eyes and fixated objects has no influence on the net retinal slip. Original traces of the fore-aft and lateral body sway with the eyes closed, eyes open in front of a wall shows the stabilizing effect of posture **(Bottom**, figure left). Viewing from a balcony with the eyes open impairs fore-aft and lateral body sway since the retinal slip of the viewed environment is subthreshold. However, with additional stationary contours of the balcony in the peripheral visual field the visual stabilization of posture is restored **(Bottom**, figure right). The influence of head movements in the frontal roll plane on postural control was not measured in the experiment. However, as stated above, retinal slip is independent of the viewing distance for head and eye movements in roll [modified from (15, 16)].

The mechanism that explains a physiological postural imbalance at heights is separate and distinct from that caused by purely cognitive reasons or anxiety as earlier researchers like Purkinje in 1820 (13) emphasized. A geometrical consideration reveals the mechanism of physiological postural imbalance at heights. In order to be visually detected, body sway must increase with increasing distance between the eyes and the stationary contrasts in the environment, because angular displacement of the surroundings on the retina is smaller, the greater the distance to them (11, 12, 14). Head sway is no longer visually detected by retinal slip (which is subthreshold) when the distance between the eyes and stationary contrasts exceeds about 3 meters. Then head and body sway rely solely on vestibular and somatosensory input. Hence, visual stabilization of posture is impaired and instability increases in the fore-aft and lateral planes. This causes an unsteadiness that increases the danger of falling (11, 14) (Figure 1).

Further analyses were performed under real stimulus conditions while subjects with visual height intolerance stood on a force-measuring platform. Major results were that open-loop control was disturbed by a higher diffusion activity, and the sensory feedback threshold for closed-loop control was lowered. This was predominantly associated with increased co-contraction of the leg muscles (15, 16). Walking in these subjects is slow and cautious, broad-based, and consists of small, flat-footed steps with less dynamic vertical oscillations of the body and head (12, 17).

The non-medical Anglo-American community uses a single term “fear of heights” to refer to a more-or-less severe visual height intolerance that, however, does not generally fulfill the criteria of the specific phobia “acrophobia.” On the basis of the above-described findings we proposed in an earlier study to distinguish between three terms in order to resolve possible confusion about physiological and pathological (psychiatric) mechanisms active during exposure to heights (18): (1) An imbalance of stance and gait at heights caused by impaired visual control. This is physiological and has no clinical relevance; (2) A visual height intolerance, which is more or less distressing and has clinical relevance for about one half of those susceptible; (3) Fear of heights or acrophobia, defined as a specific phobia in psychiatry (19, 20) which requires psychotherapy (7). In our study which was based on a questionnaire we did not differentiate between visual height intolerance and acrophobia, instead used visual height intolerance (vHI) as the umbrella term.

### Why investigate susceptibility to vHI in bilateral vestibulopathy and other vestibular disorders?

The major question of the current study, i.e., whether bilateral vestibulopathy (BVP) is a trigger of vHI (7), is related to an impaired balance in both conditions. Thus, postural instability in BVP could act as a trigger of vHI. More specifically, there were three reasons which prompted us to investigate a potential increase in susceptibility of patients with BVP to vHI:

1. The loss of vestibular function causes not only oscillopsia during locomotion, but also unsteadiness of stance and gait. This unsteadiness is partially compensated for by visual substitution of the impaired vestibular control of postural balance.
2. Visual control of balance, however, is impaired at substantial heights. When stationary targets are viewed from distant/remote heights, the retinal slip of the image falls below the threshold value at which head and body sway can no longer be detected (Figure 1).
3. Balance is further impaired by an associated sensory polyneuropathy, which is found in about 25% of the mostly elderly patients with BVP. It also occurs with associated cerebellar symptoms in 25% of these patients, (21), in part overlapping. It has been shown that the combination of BVP and polyneuropathy particularly increases instability of stance and gait, which leads to frequent falls in darkness and/or on uneven ground (22).

All three reasons make it likely that patients with BVP adjust their behavior in order to avoid experiencing visual heights, since balance control puts special demands on this condition.

The occurrence of vHI is clearly related to body position. It is strongest during erect stance, when maintaining balance is most difficult, less severe when kneeling down or sitting, and minimal or absent when the subject is lying while looking down (11). This is supported by the experiences of airplane passengers, who do not complain about having vHI.

The current study on the effects of BVP and other vestibular and functional disorders of vertigo and balance on the susceptibility to vHI is based on a standardized questionnaire applied to patients who were examined as outpatients in the German Centre for Vertigo and Balance Disorders (DSGZ).

## Methods

### Patients and data collection

A total of 150 patients with complete or asymmetrically incomplete loss of peripheral vestibular function were surveyed. Further, 481 patients with other vestibular or somatoform/functional disorders were included for comparison: vestibular migraine (*n* = 51), Menière's disease (*n* = 112), benign paroxysmal positional vertigo (*n* = 97), vestibular paroxysmia (*n* = 25), unilateral vestibulopathy (*n* = 94), and phobic postural vertigo (functional dizzines) (*n* = 102). Patients were included if they were aged 18 or above and had been referred to the DSGZ. All patients received a complete neurological, neuro-ophthalmological, and neuro-otological examination by experts in neuro-otology. Data were extracted from clinical patient records and the specific questionnaires.

### Inclusion criteria

The diagnosis of BVP was based on the patient history as well as bedside and laboratory examinations. Patients complained of unsteadiness when walking or standing, which worsened in darkness and/or on uneven ground, but had no such symptoms while sitting or lying down under static conditions. Laboratory examinations revealed a bilaterally reduced or absent angular vestibulo-ocular reflex (VOR; VOR gain <0.6 on both sides) (indicating a high frequency deficit of the VOR) and bilaterally diminished (mean peak slow-phase velocity <5 deg/s on both sides) or absent nystagmus responses to caloric irrigation (indicating a low frequency deficit). These criteria largely correspond to the recently published Consensus Document of the Classification Committee of the Bárány Society (23). The diagnosis of the other vestibular and functional disorders of vertigo and balance was also based on the criteria of the classification committee of the Bárány Society (24–29). All patients had given their informed consent.

### Exclusion criteria

Exclusion criteria were other disorders of stance and gait, cognitive impairment, psychiatric disorders, severe visual loss, chronic medication with sedatives or drug/substance abuse.

### Questionnaires

Susceptibility and characteristics of vHI were ascertained by a questionnaire containing questions to symptoms, triggers, course of the condition, and compensational behavior (30). It had been adapted and extended to include patients with BVP and other vestibular and functional disorders by adding questions on quality of life, physical and sporting activities, social functioning, avoidance behavior, and motion sickness susceptibility.

The German version of the Dizziness Handicap Inventory (DHI) was used in 49 of the 150 patients with BVP to evaluate vertigo-specific functioning (31). The DHI consists of 25 items that can be grouped into three domains representing functional, emotional and physical aspects. A total score is obtained by summing each ordinal scaled response. The total score ranges from 0-100 points; higher scores indicate a more severe handicap. The sub-domains consist of seven physical items, nine functional items, and nine emotional items.

### Statistics

Differences were tested using Student's *t*-Tests for numerical and Chi-Square Tests for categorical variables on an explorative testwise alpha level of 0.05. SAS V9.4 for Windows 10 was used for all analyses.

## Results

Of the 150 patients with a verified diagnosis of BVP (mean age 70.0, 47% females, 31% academics) vHI was reported by 29% (females 32%, males 25%; see Table 1). Of those with vHI 70% reported avoidance of climbing, hiking, stairs, darkness, cycling, swimming, or skiing (84% of those without vHI). Patients with vHI were not significantly younger (67 vs. 71 years, *p* = 0.165) and had had a significantly earlier lifetime onset of BVP (58 vs. 64 years, *p* = 0.031). Mean age for onset of vHI was 40 years.

**Table 1:** Frequency of vHI, migraine, motion sickness, anxiety, polyneuropathy, and fear or panic in patients with BVP.

		**Visual height intolerance in those with BVP**			
	**Total**	**No**		**Yes**	
	***N***	***N***	***%***	***N***	***%***
**AGE GROUP**					
<30	2	2	100.0	0	0
30–39	2	1	50.0	1	50.0
40–49	5	2	40.0	3	60.0
50–59	21	14	66.7	7	33.3
60–69	29	20	69.0	9	31.0
70–79	56	40	71.4	16	28.6
80+	35	28	80.0	7	20.0
**SEX**					
Male	79	59	74.7	20	25.3
Female	71	48	67.6	23	32.4
**MIGRAINE**					
No	125	90	72.0	35	28.0
Yes	25	17	68.0	8	32.0
**MOTION SICKNESS**					
No	130	96	73.8	34	26.2
Yes	20	11	55.0	9	45.0
**PHOBIA/ANXIETY**					
No	141	100	70.9	41	29.1
Yes	9	7	77.8	2	22.2
**POLYNEUROPATHY**					
No	59	41	69.5	18	30.5
Yes	33	26	78.8	7	21.2
Total	150	107	71.3	43	28.7

Among persons with polyneuropathy, 21% had vHI (31% of those without polyneuropathy had vHI); among persons with migraine, 32% reported vHI (28% without migraine); among persons with motion sickness, 45% reported vHI (26% without motion sickness).

Sixteen percent of all BVP patients had one or more family members with vHI (3—parents, 3—siblings, 1—children). Anxiety, panic attacks, or any phobia was present in 11% of all BVP patients. Among those with anxiety, vHI was reported by 41%. Climbing a tower was the most frequent first trigger for vHI, followed by climbing a ladder or looking out of the window of a high building; 35% (15/43) reported they avoid hiking in the mountains (this was identical in BVP patients without vHI), 9% (4/43) avoid this because of vHI. Further details on specific symptoms (such as swaying vertigo, inner agitation, trembling, diffuse sweating) concerning vHI attacks and the manifestation of BVP as well as its time-course since then are not reported here for two reasons: first, the limitation of the reliability of these details collected in a printed questionnaire, and second, because most patients with BVP cannot determine the exact date and time-course of the bilateral vestibular failure.

Patients with vHI scored higher on the DHI total (46.8 vs. 39.7), but the difference was not significant. We did not see any significant differences in instrumental measures between patients with and without vHI.

Susceptibility to vHI was higher in patients with other disorders of vertigo and balance than in BVP patients (Table 2): Prevalence of vHI was 64% in patients with phobic postural vertigo, 61% in vestibular migraine, 56% in vestibular paroxysmia, 54% in benign paroxysmal positional vertigo, 49% in unilateral vestibulopathy, and 48% in Menière's disease. As depicted in Table 3 these disorders were also associated with an increased frequency of fear or panic ranging from 20 to 37%, whereas in BVP it only amounted to 11%.

**Table 2:** Frequency of vHI in benign paroxysmal positional vertigo (BPPV), bilateral vestibulopathy (BVP), functional dizziness/phobic postural vertigo, Menière's disease, unilateral vestibulopathy (UVP), vestibular migraine, and vestibular paroxysmia.

		**Visual height intolerance**			
	**Total**	**No**		**Yes**	
	***N***	***N***	**%**	***N***	**%**
**DIAGNOSIS**					
BPPV	97	45	46.4	52	53.6
BVP	150	107	71.3	43	28.7
Functional dizziness	102	37	36.3	65	63.7
Menière's disease	112	58	51.8	54	48.2
UVP	94	48	51.1	46	48.9
Vest. migraine	51	20	39.2	31	60.8
Vest. paroxysmia	25	11	44.0	14	56.0
Total	631	326	51.7	305	48.3

**Table 3:** Frequency of fear or panic in seven disoreders of vertigo and balance (benign paroxysmal positional vertigo (BPPV), bilateral vestibulopathy (BVP), functional dizziness/phobic postural vertigo, Menière's disease, unilateral vestibulopathy (UVP), vestibular migraine, and vestibular paroxysmia.

		**Fear or Panic**			
	**Total**	**No**		**Yes**	
	***N***	***N***	**%**	***N***	**%**
**DIAGNOSIS**					
BPPV	97	76	78.4	21	21.6
BVP	150	133	88.7	17	11.3
Functional dizziness	102	64	62.7	38	37.3
Menière's disease	112	91	81.3	21	18.8
UVP	94	73	77.7	21	22.3
Vest. migraine	51	36	70.6	15	29.4
Vest. paroxysmia	25	20	80.0	5	20.0
Total	631	493	78.1	138	21.9

## Discussion

### Visual height intolerance and bilateral vestibulopathy

The survey did not support our expectation that BVP patients would have a heightened susceptibility to vHI. We found that the overall susceptibility to visual height intolerance of varying severity including acrophobia was not significantly higher than that for the general German population.

Patients with BVP indicated a current susceptibility rate of 29% (in females 32%, in males 25%), whereas the life-time prevalence in the general population is 28% (in females 32%, in males 25%) (30). The value of comparing different age groups (Table 4) is limited because of the lower random sample of the current study.

**Table 4:** Comparison of the life-time prevalence of visual height intolerance (vHI) drawn from a cross-sectional epidemiological study on 3,517 individuals (middle column, 15) and the reported susceptibility to vHI in patients with acquired BVP (right column) depicted for age groups from below 30 to above 60 years.

**Age group**	**From Huppert et al. (32)**	**Patients with BVP**
<30	29% (144/495)	0% (0/2)
30–39	28% (117/417)	50% (1/2)
40–49	31% (214/691)	60% (3/5)
50–59	33% (230/698)	33% (7/21)
> = 60	25% (304/1216)	26% (32/121)

The characteristics of the development and course of BVP did not allow us to correlate the pronounced variations in vHI susceptibility with disease onset and the severity of BVP. The onset of BVP may be abrupt as in meningitis or after intake of ototoxic antibiotics, or stepwise for each ear as in Menière's disease, or slowly progressive as in the majority of cases with degenerative or “idiopathic” etiology (21, 32). In the patients with associated polyneuropathy and/or cerebellar symptoms, conditions that further increase postural instability, we did not find an enhanced susceptibility to vHI. The specific triggers of vHI are the same in patients with and without BVP: climbing a tower was the most frequent first trigger for vHI, followed by climbing a ladder or looking out of the window of a high building.

These results are surprising, since the occurrence and severity of vHI and acrophobia critically depend on body position. Visual height intolerance is strongest during free upright stance at heights when the major concern and anxiety of the individual is to fall (11, 14). Several studies have focused on postural instability in patients with BVP, in particular if visual and/or proprioceptive substitution of the diminished/absent vestibular input is experimentally impaired (33) and also when virtual reality stimulation is used (34). Gait analysis of BVP patients walking on a pressure-sensitive carpet revealed that especially increased gait fluctuations during slow walking are most predictive of an increased fall risk (22). This study also found that a sensory polyneuropathy further critically impairs postural instability. A controlled cross-sectional study reported that the rate of recurrent fallers was 30% in patients with BVP and associated polyneuropathy (35). This increases fear of falling, deteriorates quality of life and negatively impacts on physical and social functioning (36). Our study showed that a considerable percentage avoided potentially dangerous situations, e.g., cycling, walking in the dark and hiking. Thus, contrary to our expectations patients with BVP did not exhibit an increased susceptibility to vHI. Possible explanations are discussed below in the Conclusions.

Another finding of our survey was that some patients with BVP may still experience motion sickness: 10% in BVP patients and 21% in patients with BVP and vHI. This seemingly disagrees with findings of various animal experiments in dogs and monkeys and observations in humans: namely, that motion sickness no longer occurred in any species following bilateral vestibular loss (37–39). It is conceivable that residual vestibular function in incomplete BVP may be responsible for the preserved susceptibility to motion sickness (40).

### Limitations

A study just based on filling out a printed questionnaire has several limitations as to the reliability of the data on triggers, symptoms, and time-course of vHI. The used questionnaire does not allow us to differentiate between vHI and the specific phobia fear of heights. In the meantime such a questionnaire has been developed (41) which was not available at the time of the study. Furthermore, the onset of BVP remains particularly unclear for most patients since it may be abrupt (i.e., secondary to ototoxic antibiotics) or slowly progressive and asymmetric, involving one ear in the beginning with subsequent involvement of the second ear. Therefore we concentrated on the overall frequency of vHI rather than trying to statistically correlate detailed statements provided by the questionnaire. Experimental ideas initiated by the study results would be systematic quantitative analyses of posture and gait in patients with BVP with and without vHI under natural stimulus conditions at heights at which vision can no longer substitute for the lack of vestibular information for balance control.

## Conclusions

Our finding that BVP does not increase susceptibility to vHI can be interpreted in two ways. First, one could argue that patients with this sensory deficit actively avoid exposure to heights (84% of BVP patients without vHI and 70% with vHI avoid climbing, hiking, stairs, darkness, cycling, swimming, or skiing). It is, however, very difficult to avoid stimuli when they are ubiquitous, e.g., staircases, balconies, bridges. Second, the objective postural instability is not the major trigger of an increased susceptibility to heights, but rather the irrational subjective anxiety at heights is the major trigger. It is well known that there are links between vestibular disorders, balance control, and anxiety. Based on pathways that mediate vestibular-autonomic interactions and anxiety, these links involve the parabrachial nucleus and its reciprocal interconnections with the amygdaloid nucleus, infralimbic cortex, and the hypothalamus (42). Several experiments have supported this, showing that susceptible subjects walk in a cautious way, both when visually exposed to heights as well as when only aware of heights but not visually exposed (17, 43) or during virtual reality stimulation [used in acrophobia research and treatment; (44)]. In a case-control study a representative sample of 2,012 individuals was surveyed in which acrophobia was associated with high rates of comorbid, anxious, and depressive disorders; migraine was also a significant predictor of acrophobia (45). With respect to our current study, the argument that increased anxiety is a trigger of vHI in patients with BVP is not supported by the findings of a study on psychiatric comorbidity in patients with various vestibular disorders. It revealed that anxiety/phobic disorders were less in BVP than in vestibular migraine, Menière's disease, vestibular paroxysmia, or benign paroxysmal positional vertigo (46). Our data confirm this psychiatric comorbidity (Table 3). Obviously in BVP anxiety is low because vestibular-autonomic interaction is reduced due to the lack of vestibular input. Both above-described interpretations need not be alternative explanations but may contribute to each other.

## Ethics statement

The questionnaire used in our study was part of a clinical routine assessment made after obtaining the prior written informed consent of the outpatients who presented at the dizziness unit. The evaluation of the data was completely anonymized. Ethical approval was not required for this study according to the ethical standards laid down in the 1964 Declaration of Helsinki.

## Author contributors

DH, TB, and MS conceived and designed the study, examined the patients, interpreted the data, and wrote the manuscript. EG statistically analyzed and interpreted the data, and wrote the manuscript.

### Conflict of interest statement

MS is Editor of Neuro-otology, Joint Chief-Editor of the Journal of Neurology, and section Editor of F1000. He has received speaker's honoraria from Abbott, Actelion, Auris Medical, Biogen, Eisai, GSK, Henning Pharma, Interacoustics, MSD, Otometrics, Pierre-Fabre, TEVA, UCB. He acts as a consultant for Abbott, Actelion, AurisMedical, Heel, IntraBio, and Sensorion. The remaining authors declare that there are no conflicts of interest, there exist no financial or other relationships that have influenced the work.

## References

[1] Walk RD Gibson EJ Tighe TJ Behaviour of light-and-dark-reared rats on a visual cliff. Science (1957) 126:80–81. 10.1126/science.126.3263.80-a 13442652

[2] Walk RD Gibson EG A comparative and analytical study of visual depth perception. Psychol Monogr. (1961) 75:15.

[3] Gibson EJ Riccio G Schmuckler MA Stoffregen TA Rosenberg D Taormina J. Detection of the traversability of surfaces by crawling and walking infants. J Exper Psychol Hum Percept Perform. (1987) 13:533–44 2965745 10.1037//0096-1523.13.4.533

[4] Lin YS Reilly M Mercer VS. Responses to a modified visual cliff by pre-walking infants born preterm and at term. Phys Occup Ther Pediatr. (2010) 30:66–78. 10.3109/01942630903291170 20170433

[5] De Hardt DC Visual cliff behavior of rats as a function of pattern size. Psychonom Sci. (1969) 15:268–9.

[6] Davidson PW Whitson TT. Some effects of texture density on visual cliff behavior of the domestic chick. J Comp Physiol Psychol. (1973) 84:522–6. 4745820 10.1037/h0034901

[7] Brandt T Huppert D. Fear of heights and visual height intolerance. Curr Opin Neurol. (2014) 27:111–7. 10.1097/WCO.0000000000000057 24300792

[8] Huppert D Benson J Krammling B Brandt T. Fear of heights in Roman antiquity and mythology. J Neurol. (2013) 260:2430–32. 10.1007/s00415-013-7073-1 23979100

[9] Bauer M Huppert D Brandt T. Fear of heights in ancient China. J Neurol. (2012) 259:2223–25. 10.1007/s00415-012-6523-5 22584951

[10] Darwin E Zoonomia, or The Laws of Organic Life. Vol. 1, of Vertigo, London: J Johnson (1794) p. 227–39.

[11] Brandt T Arnold F Bles W Kapteyn TS. The mechanism of physiological height vertigo. I. Theoretical approach and psychophysics. Acta Otolaryngol. (1980) 89:513–23. 6969515 10.3109/00016488009127169

[12] Brandt T Kugler G Schniepp R Wuehr M Huppert D. Acrophobia impairs visual exploration and balance during standing and walking. Ann NY Acad Sci. (2015) 1343:37–48. 10.1111/nyas.12692 25722015

[13] Purkinje JE Beiträge zur näheren Kenntnis des Schwindels aus heautognostischen Daten. Med Jahrb (1820) 6:79–125.

[14] Bles W Kapteyn TS Brandt T Arnold F. The mechanism of physiological height vertigo. II. Posturography. Acta Otolaryngol. (1980) 89:534–40. 6969517 10.3109/00016488009127171

[15] Wühr M Kugler G Schniepp R Eckl M Pradhan C Jahn K. Balance control and anti-gravity muscle activity during the experience of fear at heights. Physiol Rep. (2014) 2:e00232. 10.1002/phy2.232 24744901 PMC3966255

[16] Kugler G Huppert D Eckl M Schneider E Brandt T. Visual exploration during locomotion limited by fear of heights. PLoS ONE (2014) 9:e105906. 10.1371/journal.pone.0105906 25165822 PMC4148313

[17] Schniepp R Kugler G Wühr M Eckl M Huppert D Pradhan C et al. Quantification of gait changes in subjects with visual height intolerance when exposed to heights. Front Hum Neurosci. (2014) 8:963. 10.3389/fnhum.2014.00963 25538595 PMC4255593

[18] Brandt T Benson J Huppert D What to call “non-phobic” fear of heights? Br J Psychiatry (2012) 190:81 10.1192/bjp.190.1.81a

[19] World Health Organization The ICD-10 Classification of Mental and Behavioural Disorders. WHO, Geneva (1993). 10.1007/BF00788743 8284737

[20] APA Diagnostic and Statistical Manual of Mental Disorders: DSM-5. 5th Edn. Washington, DC: American Psychiatric Publishing (2013).

[21] Zingler VC Cnyrim C Jahn K Weintz E Fernbacher J Frenzel C et al. Causative factors and epidemiology of bilateral vestibulopathy in 255 patients. Ann Neurol. (2007) 61:524–32. 10.1002/ana.21105 17393465

[22] Schniepp R Schlick C Schenkel F Pradhan C Jahn K Brandt T et al. Clinical and neurophysiological risk factors for falls in patients with bilateral vestibulopathy. J Neurol. (2017) 264:277–283. 10.1007/s00415-016-8342-6 27878442

[23] Strupp M Kim JS Murofushi T Straumann D Jen JC Rosengren SM et al. Bilateral vestibulopathy: diagnostic criteria consensus document of the Classification Committee of the Bárány Society. J Vestib Res. (2017) 27:177–89. 10.3233/VES-170619 29081426 PMC9249284

[24] Lempert T Olesen J Furman J Waterston J Seemungal B Carey J et al. Vestibular migraine: diagnostic criteria. J Vestib Res. (2012) 22:167–72. 10.3233/VES-2012-0453 23142830

[25] Lopez-Escamez JA Carey J Chung WH Magnusson M Mandalà M Newman-Toker. Diagnostic criteria for Menière's disease. J Vestib Res. (2015) 25:1–7. 10.3233/VES-150549 25882471

[26] Von Brevern M Bertholon P Brandt T Fife T Imai T Nuti D et al. Benign paroxysmal positional vertigo: Diagnostic criteria. J Vestib Res. (2015) 25:105–17. 10.3233/VES-150553 26756126

[27] Brandt T Dieterich M Strupp M Vertigo and Dizziness: Common Complaints 2nd Edn. London: Springer (2013).

[28] Strupp M Lopez-Escamez JA Kim JS Straumann D Jen JC Carey J et al. Vestibular paroxysmia: diagnostic criteria. J Vestib Res. (2016) 26:409–15. 10.3233/VES-160589 28262641 PMC9249278

[29] Staab J Eckhardt-Henn A Horii A Jacob R Strupp M Brandt T et al. Diagnostic criteria for persistent postural-perceptual dizziness (PPPD): consensus document of the committee for the Classification of Vestibular Disorders of the Bárány Society. J Vestib Res. (2017) 27:191–208. 10.3233/VES-170622 29036855 PMC9249299

[30] Huppert D Grill E Brandt T. Down on heights? One in three has visual height intolerance. J Neurol. (2013) 260:597–604. 10.1007/s00415-012-6685-1 23070463

[31] Kurre A van Gool CJ Bastiaenen CH Gloor-Juzi T Straumann D de Bruin ED. Translation, cross-cultural adaptation and reliability of the German version of the dizziness handicap inventory. Otol Neurotol. (2009) 30:359–67. 10.1097/MAO.0b013e3181977e09 19225437

[32] Strupp M Feil K Dieterich M Brandt T. Bilateral vestibulopathy. Handb Clin Neurol. (2016) 137:235–240. 10.1016/B978-0-444-63437-5.00017-0 27638075

[33] Sprenger A Wojak JF Jandl NM Helmchen C. Postural control in bilateral vestibular failure: Its relation to visual, proprioceptive, vestibular, and cognitive input. Front Neurol (2017) 8:444. 10.3369/fneur.2017.00444 28919878 PMC5585141

[34] Chiarovano E Wang W Rogers SJ MacDougall HG Curthoys IS de Waele C. Balance in virtual reality: effect of age and bilateral vestibular loss. Front Neurol. (2017). 8:5. 10.3369/fneur.2017.00005 28163693 PMC5247457

[35] Schlick C Schniepp R Loidl V Wuehr M Hesselbarth K Jahn K. Falls and fear of falling in vertigo and balance disorders: a controlled cross-sectional study. J Vestib Res. (2016) 25:241–51. 10.3233/VES-150564 26890425

[36] Guinand N Boselie F Guyot JP Kinga H. Quality of life of patients with bilateral vestibulopathy. Ann Otol Laryngol. (2012) 121:471–77. 10.1177/000348941212100708 22844867

[37] Wang SC Chinn HJ. Experimental motion sickness in dogs. Importance of labyrinth and vestibular cerebellum. Am J Physiol. (1956) 185: 617–23. 13340001 10.1152/ajplegacy.1956.185.3.617

[38] Money KE Friedberg J. The role of the semicircular canals in causation of motion sickness and nystagmus in the dog. Can J Physiol Pharmacol. (1964) 42:793–801. 14324212 10.1139/y64-089

[39] Money KE. Motion sickness. Physiol Rev. (1970) 50:1–39. 4904269 10.1152/physrev.1970.50.1.1

[40] Dai M Raphan T Cohen B. Labyrinthine lesions and motion sickness susceptibility. Exp Brain Res. (2007) 178:477–87. 17256169 10.1007/s00221-006-0759-1 PMC3181155

[41] Huppert D Grill E Brandt T. A new questionnaire for estimating the severity of visual height intolerance and acrophobia by a metric interval scale. Front Neurol. (2017) 8:211. 10.3389/fneur.2017.00211 28620340 PMC5451500

[42] Balaban CD Thayer JF. Neurological bases for balance-anxiety links. J Anxiety Disord. (2001) 15:53–79. 10.1016/S0887-6185(00)00042-6 11388358

[43] Carpenter MG Frank JS Silcher C Peysar GW. The influence of postural threat on the control of upright stance. Exp Brain Res. (2001) 138:210–8. 10.1007/s002210100681 11417462

[44] Coelho CM Waters AM Hine TJ Wallis G. The use of virtual reality in acrophobia research and treatment. J Anxiety Disord. (2009) 23:563–74. 10.1016/j.janxdis.2009.01.014 19282142

[45] Kapfhammer HP Huppert D Grill E Fitz W Brandt T. Visual height intolerance and acrophobia: clinical characteristics and comorbidity patterns. Eur Arch Psychiatry Clin Neurosci. (2015) 265:375–85. 10.1007/s00406-014-0548-y 25262317

[46] Lahmann C Henningsen P Brandt T Strupp M Jahn K Dieterich. Psychiatric comorbidity and psychosocial impairment among patients with vertigo and dizziness. J Neurol Neurosurg Psychiatry (2015) 86:302–8. 10.1136/jnnp-2014-307601 24963122

